# Amelanotic Melanoma of the Sinonasal Region: Diagnostic Challenges

**DOI:** 10.30699/ijp.2025.2052388.3414

**Published:** 2025-08-15

**Authors:** Irianiwati Widodo, Sagung Rai Indrasari, Tri Budiarti, Naomi Yoshuantari, Ery Kus Dwianingsih

**Affiliations:** 1 *Department of Anatomic Pathology, Faculty of Medicine, Public Health and Nursing. Gadjah Mada University, Yogyakarta, Indonesia*; 2 *Sardjito General Hospital, Yogyakarta, Indonesia *; 3 *Department of Ear, Nose, and Throat (ENT), Faculty of Medicine, Public Health and Nursing, Gadjah Mada University, Yogyakarta, Indonesia*

**Keywords:** Amelanotic Melanoma, Sinonasal Melanoma, HMB45

## Abstract

**Background & Objective:**

Sinonasal melanoma is an aggressive malignancy with a poor prognosis, largely due to its propensity for local invasion and early metastasis. Diagnosis is often difficult, particularly in the absence of melanin pigmentation. Histopathological and immunohistochemical (IHC) evaluation is essential for confirmation. This report describes a diagnostically challenging case of amelanotic melanoma of the sinonasal region.

**Case Presentation:**

A 56-year-old woman presented with a 5-month history of epistaxis, facial pain, and visual impairment of the left eye. Clinical examination revealed a mass in the left nasal cavity, initially diagnosed as sinonasal carcinoma. Histopathology suggested a differential diagnosis of non-Hodgkin lymphoma (NHL) and poorly differentiated squamous cell carcinoma (SCC). Imaging demonstrated a sinonasal tumor involving the left extraconal orbital wall and paranasal sinuses. A biopsy initially raised suspicion for NHL; however, IHC staining was negative for CD45, CD20, and CD3. Similarly, negative P40 and cytokeratin excluded SCC. Strong immunoreactivity for S100, HMB45, and Melan-A established the diagnosis of amelanotic melanoma.

**Conclusion:**

Amelanotic melanoma of the sinonasal tract poses a significant diagnostic challenge due to nonspecific clinical features and lack of pigmentation. This case highlights the indispensable role of IHC in achieving a definitive diagnosis.

## Introduction

Melanoma is a neural-derived neoplasm originating from melanocytes that exhibit melanocytic differentiation. Its incidence varies across populations and is influenced by factors such as race, skin type, lifestyle, and geographic location (1). Mucosal melanoma typically arises in older adults and demonstrates a more aggressive clinical course than cutaneous melanoma. Unlike the cutaneous form, mucosal melanoma is not associated with solar exposure (2,3).

Sinonasal melanoma is rare, representing less than 1% of all head and neck tumors. It occurs more frequently in women and is most commonly diagnosed in the seventh and eighth decades of life (4). The most frequent presenting symptoms are nasal obstruction and epistaxis; however, these nonspecific features often delay diagnosis, contributing to a poor prognosis (4,5).

Amelanotic melanoma, a subtype that lacks melanin granules, presents as nonpigmented lesions. It is more frequently located in the head and neck and is less often associated with a precursor nevus. While most melanomas appear dark, amelanotic lesions may present as pink, reddish, gray, or brownish masses. This atypical presentation often leads to misdiagnosis as benign or other malignant tumors. Diagnostic delay frequently results in detection at advanced stages, when tumors are ulcerated or highly vascular, further worsening survival outcomes (6-8).

Histopathologic diagnosis of amelanotic melanoma is particularly challenging. Most sinonasal tumors display epithelioid morphology, with tumor cells arranged in sheets, exhibiting moderately pleomorphic oval to round nuclei, prominent nucleoli, and eosinophilic cytoplasm. These features may overlap with those of undifferentiated carcinomas and small round cell tumors such as non-Hodgkin lymphoma (NHL) and neuroblastoma. Immunohistochemistry remains the gold standard for accurate diagnosis. Despite the absence of melanin pigment, strong immunoreactivity for markers such as S100 and HMB-45 is highly specific for amelanotic melanoma (2,5,9).

## Case Presentation

**Fig. 1:**
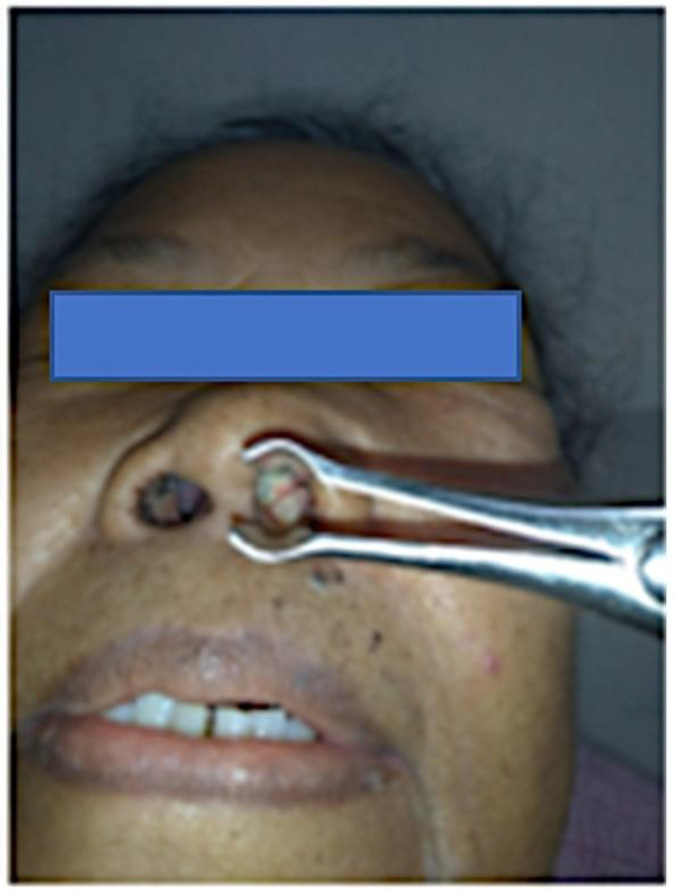
The clinical features of the sinonasal tumor include a largest diameter of 4 cm, which causes nasal obstruction. It extended to damage the wall of the left extraconal eye and involved the left maxillary sinus, as well as the ethmoidal, sphenoidal regions, and the nasopharynx.

A 56-year-old woman has been suffering from left nasal cavity obstruction, epistaxis, facial pain, and visual impairment for 5 months. The first biopsy was done outside the hospital. Histopathological diagnosis was a round blue cell tumor, with differential diagnosis of NHL and poorly differentiated Squamous Cell Carcinoma (SCC). The patient was referred to our hospital, Sardjito General Hospital, to determine a proper diagnosis and for further management. Radiological examination confirmed tumor presence in the sinonasal, 4.4x2.7x5.0 cm, extended and disrupted the wall of the left extraconal eye, left sinus maxillaries, ethmoidal and sphenoidal, as well as nasopharynx (Fig. 1). 

**Fig 2:**
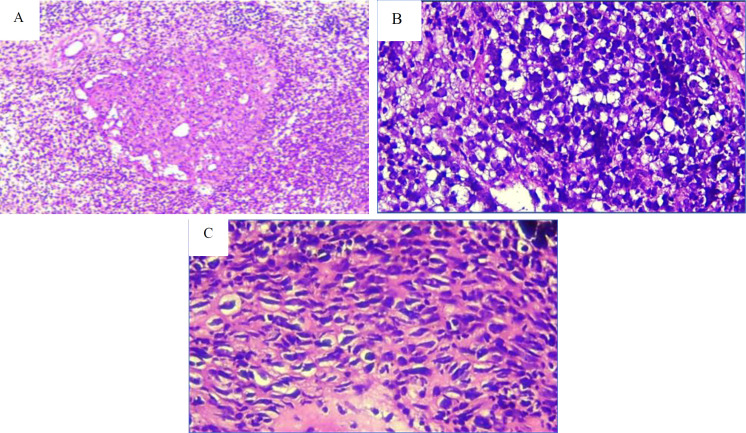
The histopathological features of the tumor include: (A) a solid growth pattern observed at 100x magnification. (B) and (C) cells that are round to spindle-shaped, featuring hyperchromatic nuclei, prominent nucleoli, and cytoplasm ranging from clear to eosinophilic, without melanin pigment (400x magnification).

A repeat biopsy was done. We received two small, white-brown colored, fragmented tissues, 1.2 x 0.6 x 0.3 cm and 0.6 x 0.6 x 0.3 cm in size. Histopathologic examination of the Hematoxylin Eosin (HE) specimen revealed a cellular tumor arranged in a solid and diffuse pattern, surrounded by necrotic and hemorrhagic areas. The tumor consists of a round-to-spindle cell tumor with hyperchromatic nuclei, prominent nucleoli, and a straightforward eosinophilic cytoplasm. No melanin pigment was found. The histopathological conclusion is similar to the biopsy from the outside hospital, NHL, and poorly differentiated SCC (Figure 2). 

**Fig 3:**
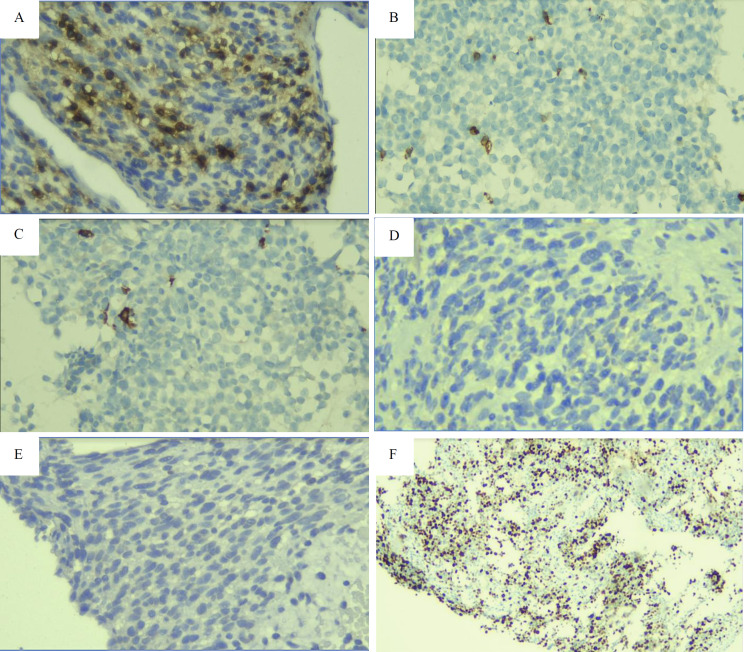
Immunohistochemical analysis showed negative results for (A) CD45, (B) CD3, and (C) CD20, which do not support a diagnosis of NHL. Additionally, the diagnosis of SCC was ruled out due to the lack of expression of (D) CK and (E) p40 (400x magnification). The high level of (F) Ki-67 indicates increased tumor proliferation (100x magnification).

However, diagnosis of NHL and SCC was then ruled out because the tumor showed negative CD45, CD20, CD3, CK, and P40 results in immunohistochemical testing (Figure 3).

**Figure 4:**
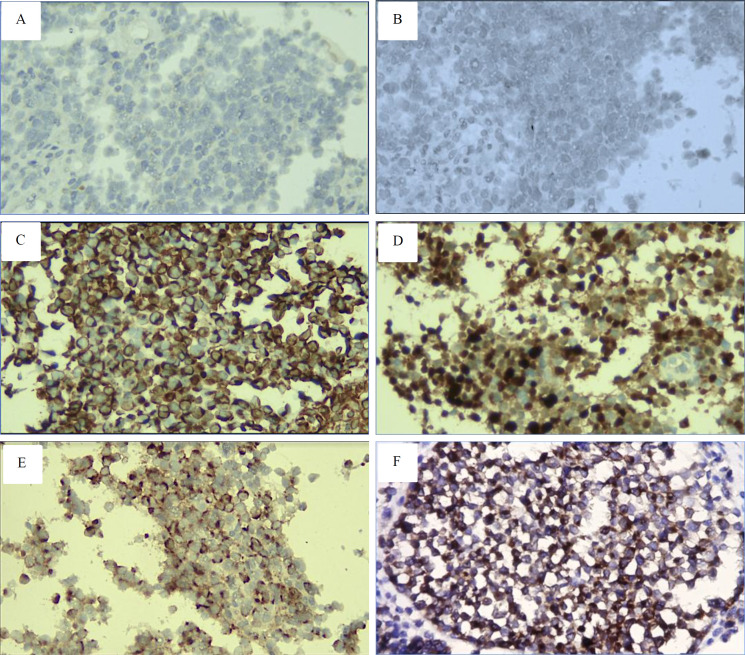
The immunohistochemical findings showed no expression of (A) synaptophysin and (B) epithelial membrane antigen (EMA), ruling out neuroblastoma and meningioma diagnoses. In contrast, strong expression of (C) Vimentin, (D) S100, (E) HMB45, and (F) Melan A supported a diagnosis of amelanotic melanoma (400x magnification).

We revisited the HE sample and proposed neural differentiation tumors such as neuroblastoma, meningioma, and amelanotic melanoma. A subsequent immunohistochemical analysis utilized antibodies targeting Vimentin, S100, synaptophysin, HMB45, Melan A, and EMA (Figure 4). 

Diagnosis of Neuroblastoma and meningioma was ruled out because tumor cells showed negative expression of synaptophysin and EMA. Surprisingly, the tumor had diffused and strong expression of Vimentin, S100, HMB45, and Melan A; therefore, a diagnosis of Amelanotic melanoma was established.

## Discussion

Amelanotic melanoma of the sinonasal is an aggressive cancer arising from melanocyte cells without melanin granules. This tumor tends to occur in older groups, and women are more affected than men. Clinical symptoms are not specific, but nasal obstruction and epistaxis are the most common signs (10). Unlike melanoma, which is usually dark, amelanotic melanoma may appear reddish with grey and brownish-colored masses, and is thus often overlooked or confused with benign lesions or other malignant tumors (2,8). The absence of melanin pigment makes the histopathological diagnosis of amelanotic melanoma difficult. The tumor may contain sarcomatous, epithelioid, or plasmacytoid cells, characterized by marked anisonucleosis and eosinophilic to basophilic cytoplasm (11,12).

Our case was a 56-year-old woman with nonspecific symptoms of epistaxis, facial pain, and visual impairment of the left eye. Radiological examination showed a large tumor (4.4x2.7x5 cm) present in the sinonasal cavity, extending and destroying the wall of the left extraconal eye, left maxillary sinus, ethmoidal, and sphenoidal sinuses, as well as the nasopharynx. The patient had been suffering from the disease for 5 months before going to the hospital, and the tumor had spread and disrupted the surrounding tissue, indicating an aggressive tumor. 

Our initial histopathological diagnosis, based on routine Hematoxylin-Eosin staining, was NHL and poorly differentiated SCC. Morphologically, the tumors were dominated by round blue cells, although some areas showed spindle and epithelioid features. Therefore, immunohistochemistry testing was carried out to confirm the diagnosis. Negative results of CD45, CD3, and CD20 ruled out the diagnosis of NHL, while negative expression of CK and p40 did not support the diagnosis of poorly differentiated SCC. Meanwhile, the proliferation index of the tumor was high (80%). Since some tumor cells morphologically resemble neural differentiation seen in neuroblastoma, meningioma, and melanoma, we continue with additional antibodies such as Vimentin, synaptophysin, EMA, S100, and HMB45. The final diagnosis of amelanotic melanoma was established when the tumor was strongly positive for vimentin, S100, and HMB45; meanwhile, diagnoses of neuroblastoma and meningioma were excluded when the tumor had a negative expression of synaptophysin and EMA. 

Diagnosis of amelanotic melanoma, in our case, requires a long time and costs a lot because of non-specific clinical manifestations and radiological and histopathological appearances. The prognosis of the tumor in our case is poor, not only because of the delay in giving therapy but also because the tumor is relatively chemo and radio resistant. The radical excision of the primary tumor has been the leading treatment choice (9,13). 

Poor prognostic factors of mucosal melanoma include depth of the local lesion, regional and distant metastasis, and vascular invasion. Regional lymph node involvement is the most important predictor of prognosis (2). The lower survival rate of this tumor is associated with older age, middle turbinate primary tumor location, advanced stage, recurrence, and distant metastasis status. The five-year survival rate of this tumor was 30.69% (4,10). The poor prognosis of sinonasal melanoma is also due to this tumor’s ability to evade the immune system due to the increased activity in cell growth and reduced activity in immune-related genes (14). Recent genetic profiling studies have shown that sinonasal melanomas are commonly driven by RAS mutations, particularly those in NRAS and C-KIT, and rarely display BRAF pathogenic variants. Detection of those genes may constitute a therapeutic target (15-17). The discovery of new therapeutics, such as immune checkpoint inhibitors (ICIs) targeting Programmed cell death protein 1 (PD-1) and cytotoxic T-lymphocyte-associated protein 4 (CTLA-4), appears promising in the treatment of mucosal melanoma (18).

## Conclusion

Diagnosing amelanotic melanoma can be challenging, time-consuming, and costly due to its nonspecific clinical signs and radiological and histopathological features. Immunohistochemical analysis plays a vital role in confirming the diagnosis. When a sinonasal tumor presents with epithelioid, sarcomatous, or lymphoid characteristics, amelanotic melanoma should be included in the differential diagnosis. This approach enables more accurate diagnosis and allows for prompt and appropriate management.
